# The impact of antimicrobial stewardship program designed to shorten antibiotics use on the incidence of resistant bacterial infections and mortality

**DOI:** 10.1038/s41598-022-04819-6

**Published:** 2022-01-18

**Authors:** Ling-Ju Huang, Su-Jung Chen, Yu-Wen Hu, Chun-Yu Liu, Ping-Feng Wu, Shu-Mei Sun, Shih-Yi Lee, Yin-Yin Chen, Chung-Yuan Lee, Yu-Jiun Chan, Yueh-Ching Chou, Fu-Der Wang

**Affiliations:** 1 Division of General Medicine, Department of Medicine, Taipei Veterans General Hospital, Taipei, Taiwan, ROC; 2 Division of Infectious Diseases, Department of Medicine, Taipei Veterans General Hospital, No. 201, Shi-Pai, Sec 2, Taipei, 11217 Taiwan, ROC; 3 School of Medicine, National Yang Ming Chiao Tung University, Taipei, Taiwan, ROC; 4 Institute of Public Health, National Yang Ming Chiao Tung University, Taipei, Taiwan, ROC; 5 Department of Oncology, Taipei Veterans General Hospital, Taipei, Taiwan, ROC; 6 Department of Infection Control, Taipei Veterans General Hospital, Taipei, Taiwan, ROC; 7 Division of Microbiology, Department of Pathology and Laboratory Medicine, Taipei Veterans General Hospital, Taipei, Taiwan, ROC; 8 College of Nursing, National Yang Ming Chiao Tung University, Taipei, Taiwan, ROC; 9 Department of Information Management, Taipei Veterans General Hospital, Taipei, Taiwan, ROC; 10 Department of Pharmacy, Taipei Veterans General Hospital, Taipei, Taiwan, ROC

**Keywords:** Health care, Medical research, Antimicrobials

## Abstract

Reassessing the continuing need for and choice of antibiotics by using an antibiotic “time out’’ program may reduce unnecessary treatment. This study aimed to explore the effect of an antibiotic stewardship program (ASP) on the antibiotics consumption, incidence of resistant bacterial infections and overall hospital mortality in a tertiary medical center during the study period 2012–2014. An ASP composed of multidisciplinary strategies including pre-prescription approval and post-approval feedback and audit, and a major “time out’’ intervention (shorten the default antibiotic prescription duration) usage was introduced in year 2013. Consumption of antibiotics was quantified by calculating defined daily doses (DDDs). Interrupted time series (ITS) analysis was used to explore the changes of antibiotics consumption before and after intervention, accounting for temporal trends that may be unrelated to intervention. Our results showed that following the intervention, DDDs showed a decreased trend in overall (in particular the major consumed penicillins and cephalosporins), in both intensive care unit (ICU) and non-ICU, and in non-restrictive versus restrictive antibiotics. Importantly, ITS analysis showed a significantly slope change since intervention (slope change p value 0.007), whereas the incidence of carbapenem-resistant and vancomycin-resistant pathogens did not change significantly. Moreover, annual overall mortality rates were 3.0%, 3.1% and 3.1% from 2012 to 2014, respectively. This study indicates that implementing a multi-disciplinary strategy to shorten the default duration of antibiotic prescription can be an effective manner to reduce antibiotic consumption while not compromising resistant infection incidence or mortality rates.

## Introduction

Antimicrobial stewardship programs (ASPs) have been proven effective for optimizing antibiotic use and minimizing adverse events, such as *Clostridium difficile* outbreaks and antibiotic resistance, and recommended as a major strategy to combat antibiotic resistance by major guidelines^1–3^. The US Centers for Disease Control and Prevention (CDC) also have summarized several core elements for inclusion in hospital ASPs since 2014^4^. A recent meta-analysis reviewed the efficacy and effect of ASPs on clinical and economic parameters and concluded that hospital ASPs result in significant decrease in antimicrobial consumption and cost, and the benefit can be higher in the critical care setting. Moreover, ASPs may improve infections due to specific antimicrobial-resistant pathogens and the overall hospital length of stay^5^.

In general, interventions to improve antibiotic prescribing practices can be conceptualized as restrictive (pre-prescription approval) or persuasive (education, post-prescription feedback and audit), as noted by Davey et al.^6^. ASP implementation may require flexibility among different sizes and types of hospital^4^. Indeed, ASPs targeted towards specific pathogens or infections and specialized environments or hospital settings have been proposed^7–10^; however, in most countries worldwide, legislation forcing health care providers to optimize their use of antimicrobial therapy has not yet been enacted^11–14^. In Taiwan, ASPs have also been implemented in several settings^15–19^. For example, pre-prescription approval is a commonly adopted strategy that has been implemented in several medical centers^15,18,19^, in some cases including modifications of post-prescription feedback/audit, such as blood culture-guided review^19^, or exemption of pre-prescription approval for intensive care unit setting^18^. Conversely, a persuasive strategy was adopted in a community-based hospital^17^.

An antibiotic “time out” prompts a reassessment of the continuing need for and choice of antibiotics when the clinical picture is clearer and more diagnostic information is available^2^. Several studies had demonstrated the effect of re-evaluation of antibiotic therapy^20^. Interestingly, the default duration of initial antibiotic prescription has rarely been evaluated in prior studies^20^. While timely reassessment of antibiotic has been used as a gold standard, this strategy can be compromised by the realities of a heavy clinical load or shortage in ID specialists. In 2013, the Centers for Disease Control, ROC (Taiwan) initiated a nationwide institutionally based project that promoted and encouraged major hospitals to implement and/or improve their ASPs. As a major member institution participating in this program, we implemented a new multi-disciplinary strategy to improve our ASP. This study aimed to evaluate the impact of an ASP, incorporating a shortened default duration of antibiotic prescription with pre-prescription approval and post-prescription feedback and audit, on the antibiotics consumption, incidence of resistant bacterial infections and overall hospital mortality.

## Material and methods

### Characteristics of institution and ASP

This study is approved by the Institutional Review Board (IRB) of Taipei Veterans General Hospital. The data was retrospectively collected containing de-identification characteristics and database of antibiotic consumption from Department of Infection Control, Taipei Veterans General Hospital. A waiver of informed consent was approved by the Taipei Veterans General Hospital IRB and all methods were carried out in accordance with the principles of the Declaration of Helsinki. Taipei Veterans General Hospital is a 2941-bed tertiary care academic medical center located in northern Taiwan. The institute contains bone marrow transplantation unit as well as solid organ transplantation units. The study period is from year 2012 to 2014. Supplementary Table S1 shows available characteristics of the study population over the study period. The distribution of several factors such as Age (including children, elderly), surgical patients, patients requiring nasogastric intubation or renal dialysis treatment, were in general similar over the study period (Supplementary Table S1).

An antimicrobial stewardship program was initially established in this institution in 1974. This antimicrobial stewardship program included formulary restriction, which required pre-prescription approval, except for patients in the intensive care unit, who often have an urgent need for antibiotics due to severe infections, and post-prescription review, feedback and audit performed by Infectious Disease (ID) physicians. This program was computer-assisted and preset a default antibiotic duration of 14 days before the initiation of a newer antimicrobial stewardship program in 2013. Most intravenous antibiotics were restricted by the pre-prescription approval, except for first and secondary generation cephalosporins and penicillins, as listed in Table 1. In 2013, a new ASP was initiated (See “Intervention” below). The core members of the new ASP include ID physicians, ID clinical pharmacists, clinical microbiologists and a computer data manager. The major intervention included in this ASP was designed to shorten the default duration of antibiotic prescription from 14 to 7 days.

**Table 1:** The list of restrictive antibiotics.

PCNs: piperacillin/tazobactam
Cephalosporine: 3rd generation (ceftriaxone, cefotaxime, flomoxef, ceftazidime, cefoperazone) and 4th generation (cefepime)
Carbapenems: ertapenem, doripenem, imipenem, meropenem
Quinolone: ciprofloxacin, levofloxacin, moxifloxacin
Glycopeptides: vancomycin, teicoplanin
Miscellulous: colistin, daptomycin, fusidate, linezolid, tigecycline

### Intervention

**Figure 1:**
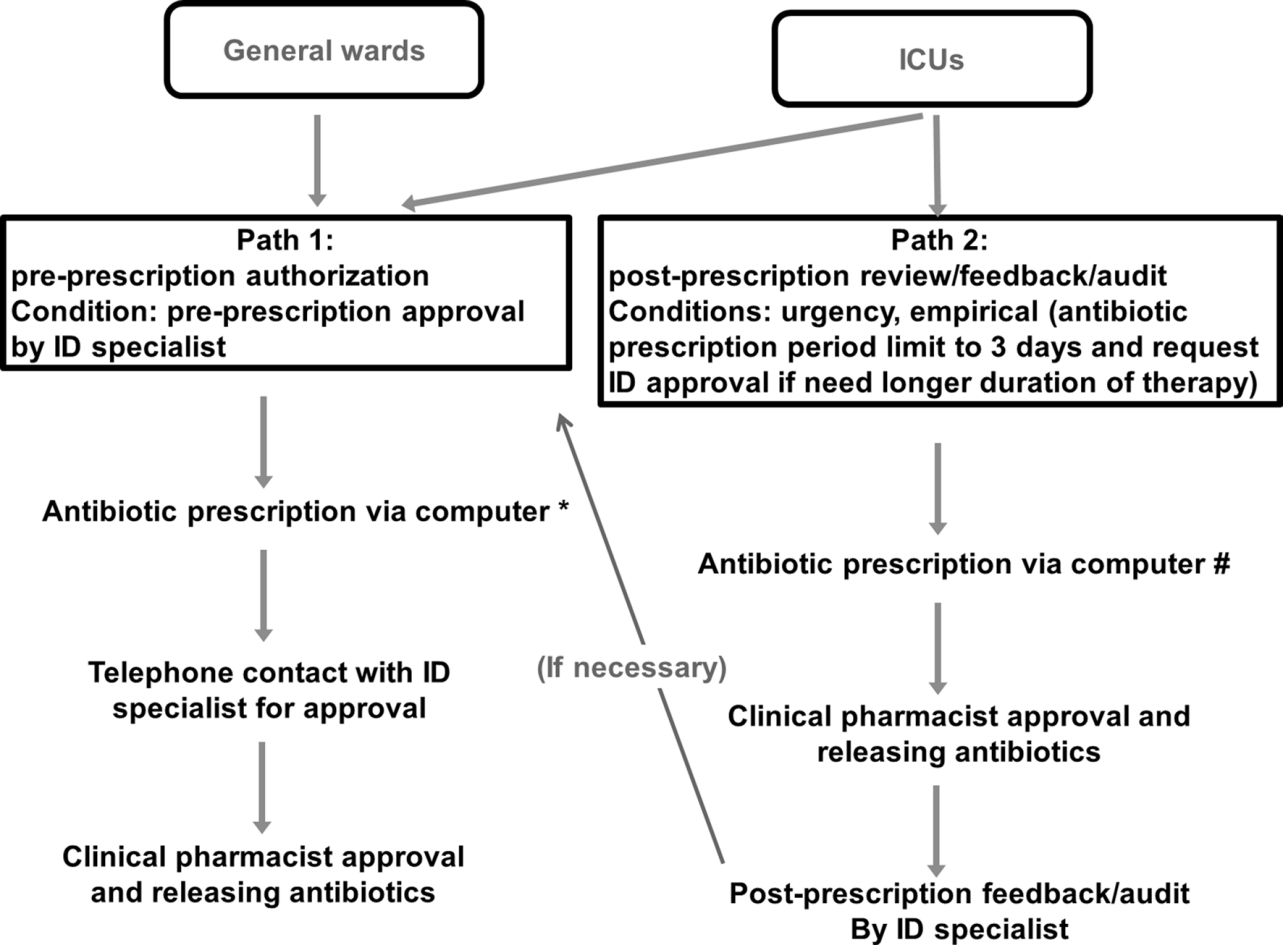
Flow chart for the restrictive antimicrobial prescription protocol. *The default duration of antibiotics prescription was 3 days for empiric therapy and 7 days for definite therapy. ^#^The default duration of antibiotics prescription was 3 days for cases requiring urgent treatment in the intensive care unit (ICU). *ID* infectious disease.

The new ASP was divided into two parts (Fig. 1). (1) In the general ward, the use of restricted antimicrobials required preapproval by ID physicians and ID clinical pharmacists. Approval was based on a discrete discussion between treating and ID physicians regarding the indications for antimicrobial use, whereas pharmacists reviewed and provided assistance in the determination of accurate dosing and scheduling of antibiotics and approved final delivery of drugs (Fig. 1, Pathway 1). For definite therapy, de-escalation or continuation of prior antibiotic therapy, the default antibiotic duration was set at 7 days. (2) In the ICU setting, use of restricted antibiotics either followed the same as protocol as was used in the general ward or was reviewed post-prescription and audited under urgent or empirical conditions (Fig. 1, pathway 2). The default duration of antibiotics for empirical or urgent treatment was 3 days, and ID physician approval was required for longer use (Fig. 1). There have been ID specialist consultants fixed to our ICU wards. ID physicians and pharmacists could access relevant real-time information via electronic medical records, including patient clinical course, laboratory and imaging results, consultation and nursing records and daily progress notes.

During the study period (year 2002–2004), several interventions were also gradually implemented. (1) Bacterial identification was gradually shifted to Matrix-Assisted Laser Desorption/Ionization Time of Flight Mass Spectrometry (MALDI-TOF) during the study period. (2) Appropriate culturing was required before hospital inpatients received empirical antibiotics if their physician prescribed restricted antibiotics for new-onset fever. (3) In the emergency room (ER), clinical physician could use restricted antibiotics without ID physician approval; however, appropriate culturing was required before ER doctors prescribed any antimicrobial agent. In critical septic patients in the ER, 2 sets of blood cultures were obtained. (4) Culture reports from the past 3 days were highlighted when providers logged into the patient’s electronic medical records. (5) In addition, reminders regarding culture reports were sent by text message to the business cell phones of attending physicians. These strategies served as reminders to providers to re-evaluate antimicrobial use after a culture report became available. Given the fact that multiple-interventions were gradually implanted during 1st quarter of 2013, we determined Apr. 2013 as a specific time point for differentiating the time period before and after the intervention of ASP for subsequent time-series analysis on the impact of ASP on antimicrobial consumption of antibiotics (see “Statistical analysis”).

### Data collection

Consumption of antibiotics was quantified by calculating defined daily doses (DDDs) according to the Anatomical, Therapeutic and Chemical Classification System/DDD System of the World Health Organization^21^, and expressed as DDD per 1000 patient-days. Nosocomial infections were defined according to the definition of the Centers for Disease Control and Prevention, and nosocomial infection density was defined as the incidence density rate of nosocomial infections (expressed as events/per 1000 patient-days.)

### Quality indicators

To evaluate the safety of this program, selective quality indicators were monitored by infection control nurses, including the rate of patient mortality, incidence of healthcare-associated infections (HAI) and detection rate of healthcare-associated drug-resistant organism including vancomycin-resistant enterococci (VRE), carbapenem-resistant *Acinetobacter baumannii* (CRAB), carbapenem-resistant *Pseudomonas aeruginosa* (CRPA), carbapenem-resistant *Escherichia coli* (CREC) and carbapenem-resistant *Klebsiella pneumoniae* (CRKP).

### Statistical analysis

Linear regression analyses were used to examine the trends in seasonal antibiotic consumption, mortality, incidence of HAIs, and detection of HA-MDROs before and after the intervention. Pearson’s correlation coefficients were applied to determine the relationships between the program implementation and trends in antibiotic consumption. Interrupted time series (ITS) analysis was adopted to account for temporal trends of antibiotics consumptions and to evaluate longitudinal impact of the introduction of ASP. ITS analysis is an approach for evaluating the effectiveness of population-level health interventions that have been implemented at a clearly defined timepoint^22,23^. The trends in antibiotics consultation (DDD per 1000 patient-days) before and after the introduction of the ASP in Apr 2013 were compared by calculating the slope change. We used the SPSS TSmodel which applies an autoregressive integrated moving average (ARIMA) model for time series analysis, detail of which has been described by others^24^. A p value < 0.05 was considered statistically significant. Analyses were performed using SPSS version 19.0 (IBM Corp, Armonk, NY).

## Results

**Figure 2:**
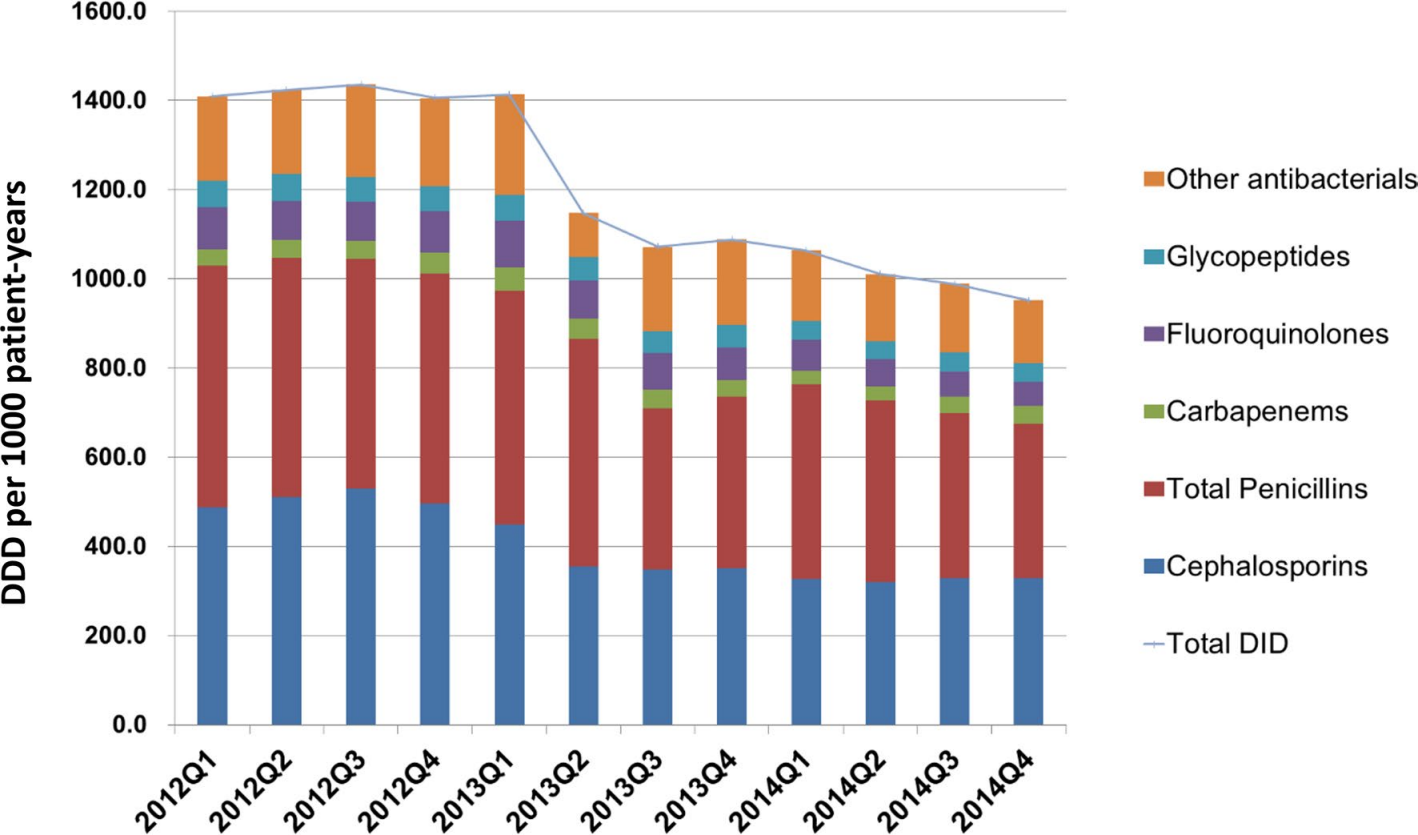
Total antimicrobial consumption of antibiotics before and after the intervention.

**Figure 3:**
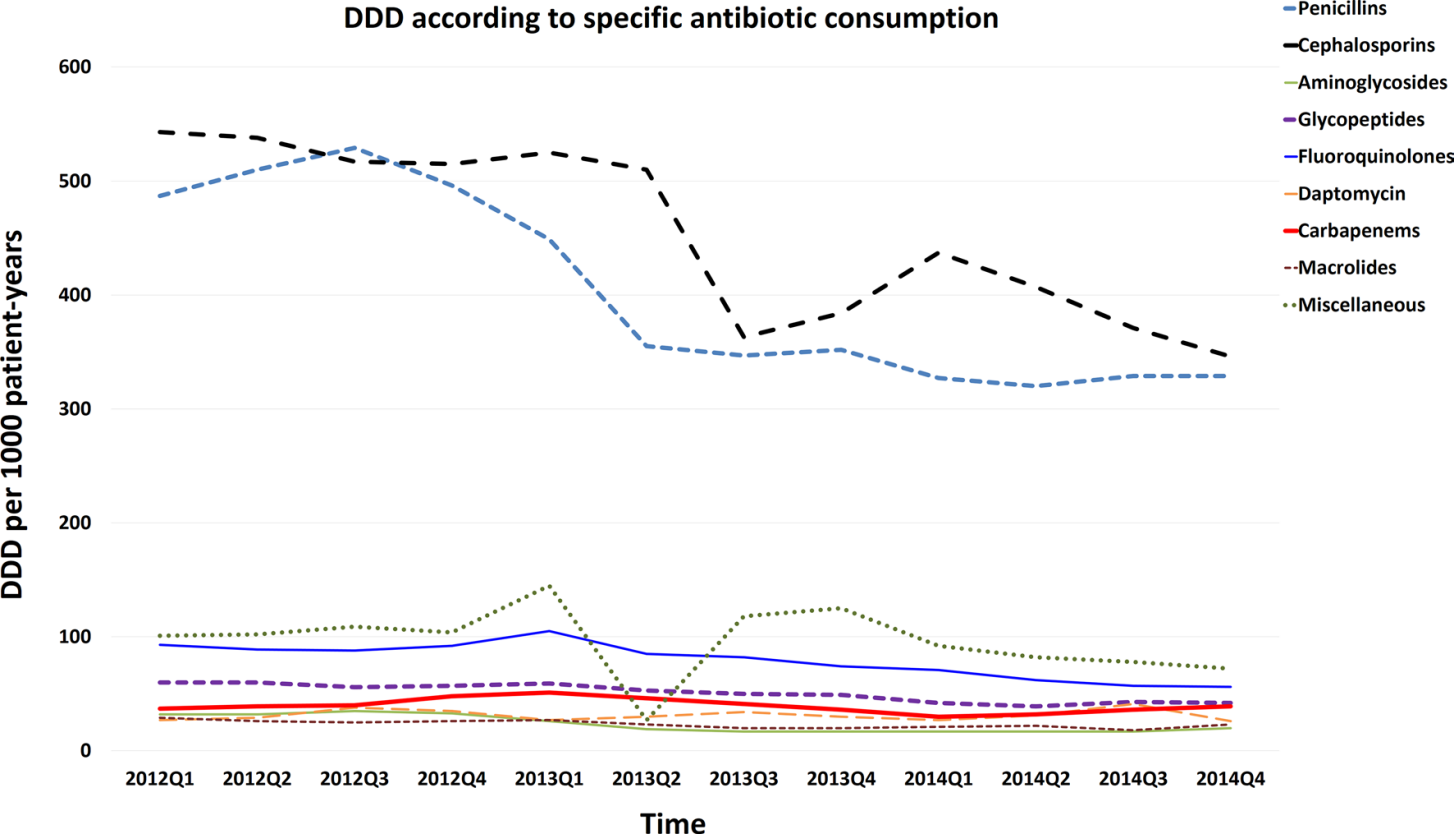
Trends of antibiotic consumption according to specific antibiotics. The major consumed antibiotics showed more prominent trend in DDD decline, whereas carbapenems, although were relatively low consumed, did not show a decrease trend (red line).

**Figure 4:**
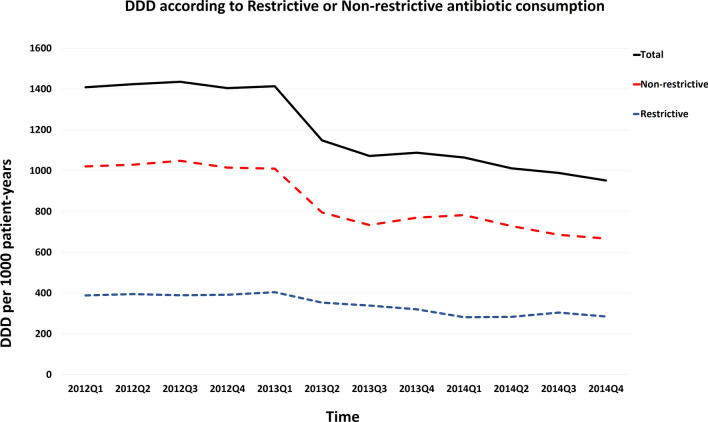
Trends of antibiotic consumption according to restrictive or non-restrictive antibiotics. Both restrictive and non-restrictive antibiotics showed a decreased trend of antibiotic consumption through the study period.

**Figure 5:**
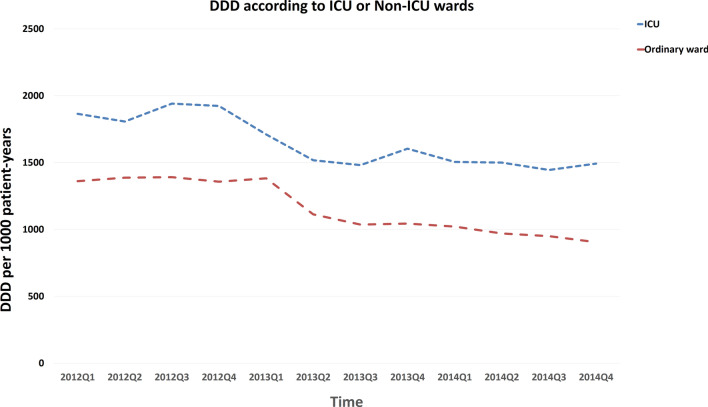
Trends of antibiotic consumption according to intensive care unit (ICU) and non-ICU ward. Both ICU and non-ICU wards showed a decreased trend of antibiotic consumption through the study period.

Before the intervention, seasonal antimicrobial consumption of all antibacterial agents ranged between 1436 to 1405 DDD/1000 patient days. There was a numerical decrease in total antibiotic consumption following the second quarter of 2013 (Fig. 2). Trends in total antimicrobial consumption are shown in Fig. 2. A trend of decreased antibiotic consumption were observed in several specific types of antibiotics, including major consumed penicillins and cephalosporins, and minor consumed fluoroquinolones, as shown in Fig. 3 (and Supplement Table S2). Because newer penicillins, new quinolones and newer (3rd or beyond) generation cephalosporoin were restrictive antibiotics (Table 1), we also analyzed the trend of antibiotics consumption according to restrictive or non-restrictive antibiotics and found both classes showed a decrease trend (Fig. 4 and Supplement Table S3). Since the ASP intervention was divided according to ICU versus non-ICU settings (Fig. 1), we found that both antibiotic consumption in ICU and non-ICU showed a decreased trend through the study period (Fig. 5 and Supplement Table S4).

There are four types of carbapenems used in our hospital: ertapenem, imipenem, meropenem and doripenem. Trends in the consumption of these carbapenems did not show a declination during the study period and notably the absolute consumption (DDDs) of either group 1 or group 2 carbapenems was low (Fig. 3, Supplement Fig. S1 and Table S5).

The annual hospital-acquired infection density rates during study period were 3.37, 3.34 and 3.43 per 1000 patient-days in 2012, 2013, and 2014, respectively. The annual detection rates of drug-resistant pathogens are shown in Table 2. The detection rates of CRAB were 59% in 2012, 59.6% in 2013 and 54.5% in 2014. The detection rates of CREC were 0.8%, 1.8% and 2.2% in 2012, 2013, and 2014, respectively. The detection rates of CRKP were 15.6%, 20.7% and 16.7% in 2012, 2013, and 2014, respectively, and the detection rates of VRE and CRPA were 29.7%, 32.0% and 32.2% and 10.6%, 16.7% and 13.4% during 2012, 2013, and 2014, respectively. There were no significant changes in the incidence rate of hospital-acquired infections or the detection rate of resistant pathogen during the study period. The overall annual mortality rates in the hospital was 3.0% (2995/99,865), 3.1% (3053/98,745), 3.1% (3015/98,210) in 2012, 2013, and 2014, respectively. There was no significant change in mortality rate during the study period.

**Table 2:** The proportion of drug resistant pathogens in nosocomial infections.

Pathogens	Before intervention	After intervention			
	Year 2012	Year 2013		Year 2014	
	n/N	Proportion (%)	*p* value (2013 v 2012)	Proportion (%)	*p* value (2014 v 2012)
CRAB	114/278 (59%)	162/272 (59.6%)	0.945	102/187 (54.5%)	0.618
CREC	4/499 (0.8%)	9/492 (1.8%)	0.160	11/504 (2.2%)	0.075
CRKP	63/403 (15.6%)	73/353 (20.7%)	0.133	45/269 (16.7%)	0.747
VRE	89/300 (29.7%)	85/266 (32.0%)	0.668	88/273 (32.2%)	0.629
CRPA	32/302 (10.6%)	43/258 (16.7%)	0.066	32/238 (13.4%)	0.367
Mortality	3.00%	3.09%		3.07%	

CRAB, carbapenem-resistant *Acinetobacter baumannii*; CREC, carbapenem-resistant *Escherichia coli*; CRKP, carbapenem-resistant *Klebsiella pneumoniae*; VRE, vancomycin-resistant enterococci; CRPA, carbapenem-resistant *Pseudomonas aeruginosa.*

### Interrupted time series analysis of the impact of ASP on antibiotic use

**Figure 6:**
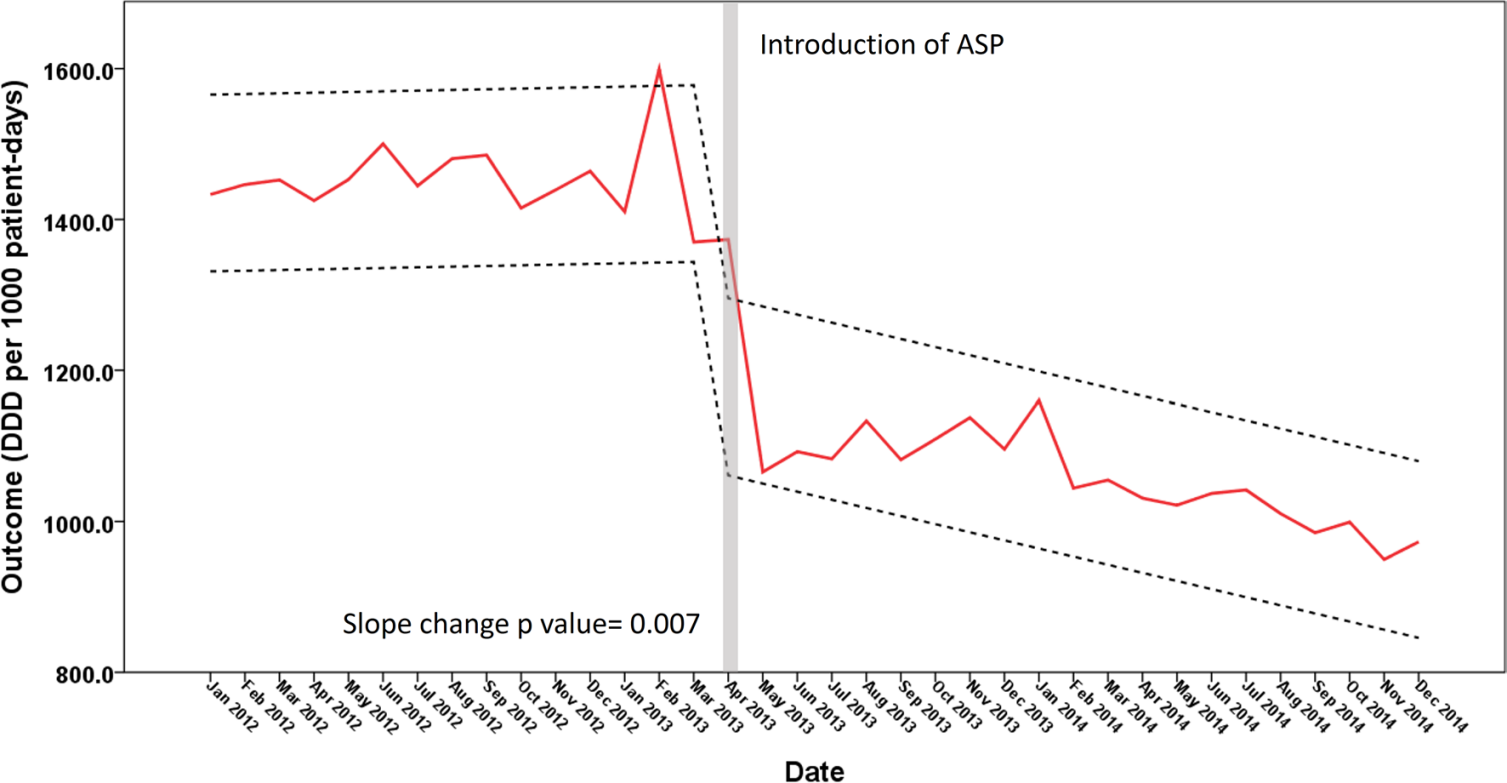
Interrupted time-series analysis of the impact of antimicrobial stewardship program implantation on antimicrobial consumption of antibiotics. Slope and level change model is used. Refer to “Material and methods” for detail. p value for slope change model is 0.007. Red solid line: predicted trend based on regression model. Black dash lines: upper and lower control limit.

Since multiple-interventions were gradually implanted during 1st quarter of 2013, we determined Apr. 2013 as a specific time point for differentiating the time period before and after the intervention of ASP for subsequent time-series analysis on the impact of ASP on antimicrobial consumption of antibiotics. As shown in Fig. 6, the slope change before and after the intervention shows a significant decrease, suggesting a gradual decrease in antibiotic consumption following time trends (p = 0.007). However, more than 6 years have elapsed since the major intervention in 2013 Q1 and study period, to explore whether there was a relapse or maintenance or even amelioration of the initial antibiotic consumption trend by intervention, we included 2 more years (2015 and 2016) of DDDs and performed ITS analysis again (Supplement Fig. S2). As shown in Supplement Fig. S2, the slope change of antibiotic consumption remained significant (slope p value 0.047), and a significant level change (level change p value < 0.001) of antibiotics consumption was also noted.

## Discussion

The ITS analysis confirmed the impact of ASP implantation on the antimicrobial consumption, showing a significant slope change (in decreasing direction) on antibiotics use following time trends during the study period (Fig. 6), and the effect of ASP seemed to sustain for at least 2 more years (Supplement Fig. S2). This decrease in antibiotic consumption may also have been contributed to by several other factors in addition to the shortened default antibiotics prescription duration. First, there was a decline in seasonal influenza pneumonia in Taiwan in 2013^25^. According to data from the Taiwan CDC, a total of 1595 critical influenza cases were identified in 2012; the number of critical cases declined to 965 in 2013 but increased to 1721 in 2014^25^. Critical influenza patients were often hospitalized and had diseases complicated by secondary bacterial infections^26,27^, and these factors might have resulted in additional antibiotic usage^28^. Therefore, the significant decline observed in the number of critical influenza cases might have also partially contributed to reduce concomitant antibiotic consumption in 2013. In addition, during the period in which the new ASP was implemented, culture report reminders were sent to physician electronic devices, and the clinical application of MALDI-TOF may have facilitated decision-making in attending physicians; these factors may have made de-escalated and targeted (narrow spectrum) antimicrobial therapy possible. The inclusion of ID clinical pharmacists in decision-making may have also contributed to decreased antimicrobial consumption.

In current study most antibiotics consumption (except for carbapenems) declined after our intervention with shortened default antibiotic prescription period (Figs. 2, 3, 4, 5). Our results differed from the “squeezing the balloon” phenomenon, a phenomenon which illustrates that successful restriction of a single class of antibiotics may result in increased consumptions of other unrestricted classes of antibiotics^29^. It is likely that implementation of an ASP applicable to a broad class of antibiotics may reduce the antibiotic selection pressure.

The trend of carbapenem consumption was not changed significantly by the intervention (Fig. 3, Supplement Fig. S1 and Table S5). The relatively low usage of carbapenems (either group 1 or group 2) may reflect the institutional attitude of judicious antibiotic usage counteracting with the emergence of important nosocomial pathogens that are sensitive to group 2 carbapenems but relatively resistant to group 1 carbapenems^30,31^. It is worthy of further study to observe the long-term trend of carbapenem consumption in association with resistant strains in our institute.

Our data showed that a shortened default antibiotics prescription duration did not compromise crude in-hospital mortality. Shortening of the number of default days pushes clinical prescribers to re-evaluate the patient; whereas priorly the clinical physician usually did not de-escalate therapy or discontinued unnecessary drugs. On the other hand, shortening the default duration of antibiotics seemed to not affect the rates of major resistant infections (Table 2). Notably, the major resistant pathogens posing a threat in our institute were CRAB and VRE species (Table 2), and controlling these infections also requires strict measures such as standard and contact barrier precautions^32,33^. Although many studies of ASPs have shown reductions in resistant infections, a few studies have demonstrated opposing results. A Brazilian study identified a higher rate of resistant infections following implementation of a bundled ASP (clinical pharmacist chart reviews, discussion with microbiologists and infectious disease physicians, local education and continuous follow-up) when compared to the rates associated with the conventional ASP (clinical pharmacist chart reviews and discussion with infectious disease physicians)^34^. In our study, urgent use of restricted antibiotics with a post-prescription audit was allowed in ICU settings (Fig. 1); this strategy might have increased antibiotic selection pressure^35,36^. Moreover, the period during which the new ASP (shortened-default antibiotic prescription duration) was implemented was relatively short (2 years), and it is possible that a longer duration is needed to see a reduction in resistant infections.

Our study has some limitations. First, our data were obtained by periodically measurement of the antibiotics consumption in the entire medical center, the patient characteristics and populations may change overtime therefore it is difficult to compare patients’ characteristics given the fluctuation of population over time. Moreover, there is lack of details regarding numbers of patients in ICU, days of ICU, as well as type of nosocomial infections in ICU. Our interventions may have less impact on antibiotics consumption due to urgent use of antibiotics and complexity of disease status as well as common emergence of resistance strains in ICU.

In summary, the implementation of a multi-disciplinary strategy to shorten the default duration of antibiotic prescriptions was effective at reducing antibiotic consumption while not compromising the rates of resistant infections or mortality.

## Supplementary Information

Supplementary Information.
