# Supplementary material for: The impact of antimicrobial stewardship program designed to shorten antibiotics use on the incidence of resistant bacterial infections and mortality
Source: Sci Rep. 2022 Jan 18;12:913. doi: 10.1038/s41598-022-04819-6 (PMC8766441; doi:10.1038/s41598-022-04819-6)
Supplement: Supplementary file 1 — Supplementary Information. [file 41598_2022_4819_MOESM1_ESM.docx]

**The impact of antimicrobial stewardship program designed to shorten antibiotics use on the incidence of resistant bacterial infections and mortality**

**Short title:** shortened default antibiotics prescription duration

Ling-Ju, Huang^1,2,3^, Su-Jung Chen^2,3,4^, Yu-Wen Hu^3,5^, Chun-Yu Liu^5^, Ping-Feng Wu^2,3^, Shu-Mei Sun^6^, Shih-Yi Lee^7^, Yin-Yin Chen^6,8^**,** Chung-Yuan Lee^9^**,** Yu-Jiun Chan^4,7^, Yueh-Ching Chou^10^, Fu-Der Wang^2,3,*^

^1^Division of General Medicine, Department of Medicine, Taipei Veterans General Hospital, Taipei, Taiwan

^2^Division of Infectious Diseases, Department of Medicine, Taipei Veterans General Hospital, Taipei, Taiwan

^3^School of Medicine, National Yang Ming Chiao Tung University, Taipei, Taiwan

^4^ Institute of Public Health, National Yang Ming Chiao Tung University, Taipei, Taiwan

^5^Department of Oncology, Taipei Veterans General Hospital, Taipei, Taiwan

^6^Department of Infection Control, Taipei Veterans General Hospital, Taipei, Taiwan

^7^Division of Microbiology, Department of Pathology and Laboratory Medicine, Taipei Veterans General Hospital, Taipei, Taiwan

^8^College of Nursing, National Yang Ming Chiao Tung University, Taipei, Taiwan

^9^Department of Information Management, Taipei Veterans General Hospital, Taipei, Taiwan

^10^Department of Pharmacy, Taipei Veterans General Hospital, Taipei, Taiwan

* Correspondence to Fu-Der Wang, MD.

Division of Infectious Disease, Department of Medicine, Taipei Veterans General Hospital, No. 201, Shi-Pai, Sec 2, Taipei, 11217, Taiwan, ROC

e-mail: [fdwang@vghtpe.gov.tw](mailto:fdwang@vghtpe.gov.tw)

TEL: +886-2-28757494

Fax: +886-2-28730052

**Supplementary Table S1.** The characteristics of patients admitted to Taipei Veterans General Hospital according to year period (2012-2014)

| Year | 2012 | | 2013 | | 2014 | |
| --- | --- | --- | --- | --- | --- | --- |
|  | Jan-June | Jul-Dec | Jan-June | Jul-Dec | Jan-June | Jul-Dec |
| Total numbers of admission | 47772 | 49485 | 47619 | 48349 | 46681 | 48888 |
| Age, years  (Mean ± SD) | 57.0 ±21.6 | 56.7 ±21.6 | 57.2 ±21.4 | 56.8 ±21.5 | 57.3 ±21.4 | 56.9 ±21.2 |
| Admission days  (Mean ± SD) | 9.3 ±19.8 | 9.4 ±35.4 | 9.4 ±41.0 | 9.3 ±33.4 | 9.2 ±32.5 | 9.2 ±32.4 |
| Male  No. (%) | 25152  (52.7%) | 25906  (52.4%) | 25227  (53.0) | 25542  (52.8%) | 24370  (52.2%) | 25007  (51.2%) |
| Age ≤14 years  No. (%) | 2043  (4.3%) | 2179  (4.4%) | 1965  (4.1%) | 2079  (4.3%) | 1886  (4.0%) | 1910  (3.9%) |
| Age ≥65 years  No. (%) | 18269  (38.2%) | 18410  (37.2%) | 17756  (37.3%) | 17815  (36.8%) | 17542  (37.6%) | 17785  (36.4%) |
| Surgery  No. (%) | 14850  (31.1%) | 15568  (31.5%) | 14553  (30.6%) | 15526 (32.1%) | 14630  (31.3%) | 16152  (33.0%) |
| Renal dialysis  No. (%) | 1338 (2.8%) | 1220 (2.5%) | 1258 (2.6%) | 1252 (2.6%) | 1250  (2.7%) | 1202  (2.5%) |
| Nasogastric tube intubation, No. (%) | 4222  (8.8%) | 4212 (8.5%) | 4286  (9.0%) | 4182  (8.6%) | 4352  (9.3) | 4681  (9.6%) |

**Supplementary Table S2**: Distribution of antibiotics usage before and after intervention ( DDD/1,000 patient-days)

|  | Before intervention | | | | | After intervention | | | | | | |
| --- | --- | --- | --- | --- | --- | --- | --- | --- | --- | --- | --- | --- |
|  | 2012 | | | | 2013 | | | | 2014 | | | |
| Antibiotics | Q1 | Q2 | Q3 | Q4 | Q1 | Q2 | Q3 | Q4 | Q1 | Q2 | Q3 | Q4 |
| Penicillins | 487 | 510 | 529 | 496 | 449 | 355 | 347 | 352 | 327 | 320 | 329 | 329 |
| Cephalosporins | 543 | 538 | 517 | 515 | 525 | 510 | 363 | 384 | 437 | 407 | 371 | 346 |
| Aminoglycosides | 32 | 32 | 35 | 33 | 26 | 19 | 17 | 17 | 17 | 17 | 17 | 20 |
| Glycopeptides | 60 | 60 | 56 | 57 | 59 | 53 | 50 | 49 | 42 | 39 | 43 | 42 |
| Fluoroquinolones | 93 | 89 | 88 | 92 | 105 | 85 | 82 | 74 | 71 | 62 | 57 | 56 |
| Daptomycin | 27 | 29 | 38 | 35 | 27 | 30 | 34 | 30 | 27 | 31 | 41 | 26 |
| Carbapenems | 37 | 39 | 40 | 48 | 51 | 46 | 41 | 36 | 30 | 32 | 36 | 39 |
| Macrolides | 29 | 26 | 25 | 26 | 27 | 23 | 20 | 20 | 21 | 22 | 18 | 23 |
| Miscellaneous | 101 | 102 | 109 | 104 | 145 | 27 | 118 | 125 | 92 | 82 | 78 | 72 |
| Total | 1409 | 1424 | 1436 | 1405 | 1414 | 1148 | 1072 | 1088 | 1064 | 1011 | 989 | 952 |

Miscellaneous: Sulbactam, Metronidazole, Clindamycin, Colistin, Tetracycline (including Tigercycline), Sulfonamides, Linezolid,

Q1, Q2, Q3, Q4: 1^st^, 2^nd^, 3^rd^ and 4^th^ quarter of the year

**Supplementary Table S3.** The antimicrobial consumption according to restrictive and nonrestrictive antibiotics (DDD/1,000 patient-days)

|  | Restrictive | | | Non-restrictive | | |  | Total |  |
| --- | --- | --- | --- | --- | --- | --- | --- | --- | --- |
|  | 2012 | 2013 | 2014 | 2012 | 2013 | 2014 | 2012 | 2013 | 2014 |
| Q1 | 388 | 404 | 281 | 1021 | 1010 | 782 | 1409 | 1414 | 1064 |
| Q2 | 395 | 353 | 283 | 1029 | 795 | 728 | 1424 | 1148 | 1011 |
| Q3 | 389 | 338 | 304 | 1048 | 733 | 685 | 1436 | 1072 | 989 |
| Q4 | 391 | 320 | 285 | 1015 | 769 | 667 | 1405 | 1088 | 952 |

**Supplementary Table S4.** Antimicrobial consumption (DDD/1,000 patient-days) in ICU and ordinary wards

|  | 2012 | | | | 2013 | | | | 2014 | | | |
| --- | --- | --- | --- | --- | --- | --- | --- | --- | --- | --- | --- | --- |
|  | Q1 | Q2 | Q3 | Q4 | Q1 | Q2 | Q3 | Q4 | Q1 | Q2 | Q3 | Q4 |
| ICU | 1865 | 1808 | 1942 | 1924 | 1711 | 1518 | 1482 | 1604 | 1506 | 1501 | 1445 | 1493 |
| Ordinary ward | 1361 | 1388 | 1391 | 1358 | 1383 | 1113 | 1037 | 1045 | 1022 | 971 | 950 | 905 |

Q1, Q2, Q3, Q4: 1^st^, 2^nd^, 3^rd^ and 4^th^ quarter of the year

**Supplementary Table S5:** Consumption of carbapenem according to groups (DDD/1,000 patient-days)

|  | 2012 | | | | 2013 | | | | 2014 | | | |
| --- | --- | --- | --- | --- | --- | --- | --- | --- | --- | --- | --- | --- |
|  | Q1 | Q2 | Q3 | Q4 | Q1 | Q2 | Q3 | Q4 | Q1 | Q2 | Q3 | Q4 |
| Group 1 | 12 | 13 | 13 | 16 | 14 | 12 | 12 | 11 | 10 | 12 | 13 | 16 |
| Group 2 | 25 | 26 | 27 | 32 | 37 | 34 | 29 | 26 | 21 | 20 | 22 | 24 |
| Total Carbapenems | 37 | 39 | 40 | 48 | 51 | 46 | 41 | 36 | 30 | 32 | 36 | 39 |

Group 1: Ertapenem broad spectrum carbapenems, with limited activity against non-fermentative gram-negative bacilli, that is particularly suitable for community acquired infections,.

Group 2 Imipenem, meropenem and doripenem, broad spectrum carbapenems with activity against non-fermentative gram-negative bacilli, that are particularly suitable for nosocomial infections ^1^.

Q1, Q2, Q3, Q4: 1^st^, 2^nd^, 3^rd^ and 4^th^ quarter of the year


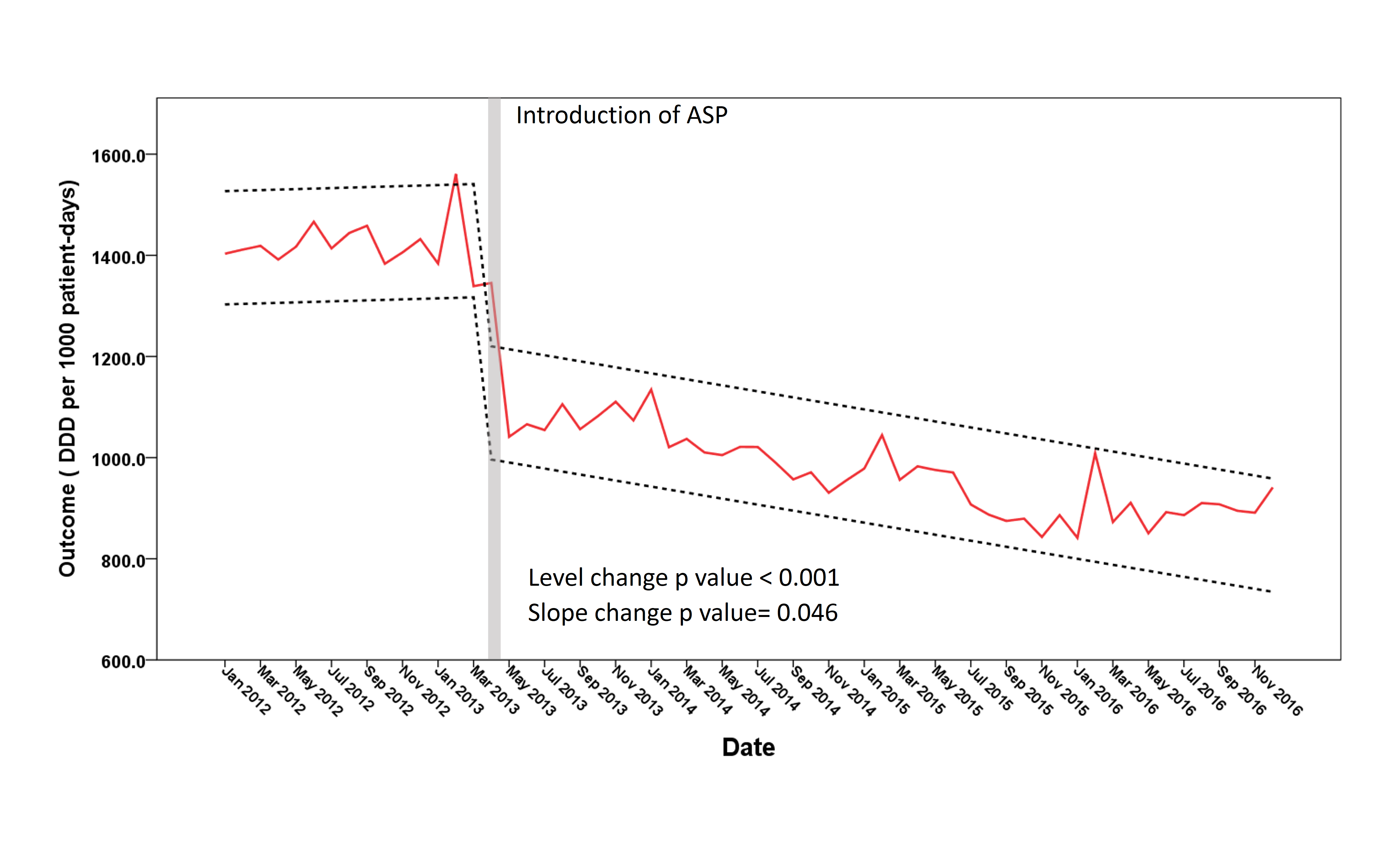


**Figure S1. Interrupted time-series (ITS) analysis of the impact of antimicrobial stewardship program implantation on antimicrobial consumption of antibiotics (From 2012 to 2016).**

Slope and level change model is used. Refer to Method for detail. When ITS analysis include 2 more subsequent years (2015-2016), there is also a significant level change (level change p value < 0.001) of antibiotics consumption in addition to the previous noted significant slope change (refer to Figure 6).

ASP, antibiotic stewardship program; Red, center line; Black-dash denotes LCL, lower control limit and UCL, upper control limit


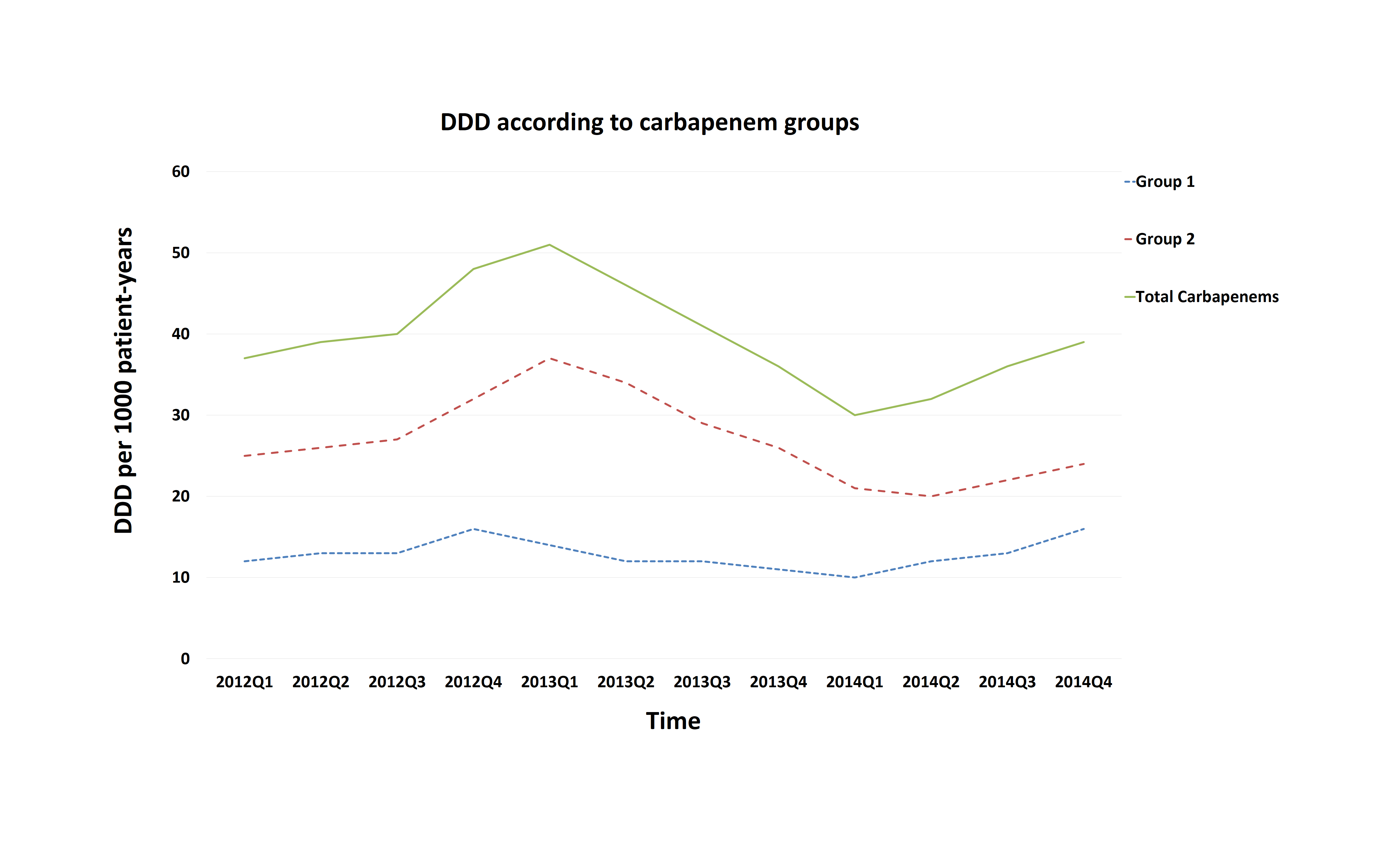


**Figure S2. Trends of antibiotic consumption according to carbapenem groups**

Neither group 1 nor group 2 Carbapenem consumption showed a decreased trend.

Group 1: Ertapenem broad spectrum carbapenems, with limited activity against non-fermentative gram-negative bacilli, that is particularly suitable for community acquired infections. Group 2: Imipenem, meropenem and doripenem, broad spectrum carbapenems with activity against non-fermentative gram-negative bacilli, that are particularly suitable for nosocomial infections.

Reference in Supplementary material

1 Shah, P. M. & Isaacs, R. D. Ertapenem, the first of a new group of carbapenems. *The Journal of antimicrobial chemotherapy* **52**, 538-542, doi:10.1093/jac/dkg404 (2003).
